# Supplementary material for: Pyridoxamine Limits Cardiac Dysfunction in a Rat Model of Doxorubicin-Induced Cardiotoxicity
Source: Antioxidants (Basel). 2024 Jan 17;13(1):112. doi: 10.3390/antiox13010112 (PMC10812466; doi:10.3390/antiox13010112)
Supplement: Supplementary file 1 [file antioxidants-13-00112-s001.zip › antioxidants-2770529-supplementary.pdf]

Supplementary Tables

Table S1: Primer sequences used for qPCR.

Gene	Forward primer	Reverse primer	Accession number
RAGE	TGGAAACTGAACACAGGAAGGA	GGAGTGAACCATTGGGGAGG	NM_053336.2
IL-6	TAGTCCTTCCTACCCCAACTTCC	TTGGTCCTTAGCCACTCCTTC	NM_012589.2
Bcl-2	CTTCTCTCGTCGCTACCGTC	GAAGAGTTCCTCCACCACCG	NM_016993.2
Bax	ACACCTGAGCTGACCTTGGA	TATCCACATCAGCAATCATCCTCT	NM_017059.2
Tp53	CATGCCCTGTGCAGTTGTG	AGCCAGGCCGTCACCATC	NM_030989.3
Casp3	ACTACTGCCGGAGTCTGACT	TAACCGGGTGCGGTAGAGTA	NM_012922.2
Col1a2	GCCAAGAATGCATACAGCCG	GACACCCCTTCTGCGTTGTA	NM_053356.2
Col3a1	AACTGGAGCACGAGGTCTTG	CGTTCCCCATTATGGCCACT	NM_032085.1
Tgfb1	ACCGCAACAACGCAATCTATG	GCACTGCTTCCCGAATGTCT	NM_021578.2
Lox	AGCTGCCACCAACATTACCA	GGGACTCAACCCCTGTGTG	NM_001414003.1
Nox2	ATGCTGATCTTGCTGCCAGT	AATGGCCGTGTGAAGTGCTA	NM_023965.2
Nox4	TCATGGATCTTTGCCTGGAGGGTT	AGGTCTGTGGGAAATGAGCTTGGA	NM_053524.1
Sod1	GCTTCTGTCGTCTCCTTGCT	CACAACTGGTTCACCGCTTG	NM_017050.1
Sod2	AGCTGCACCACAGCAAGCAC	TCCACCACCCTTAGGGCTCA	NM_017051.2
Gpx1	CAATCAGTTCGGACATCAGGAGAA	CTCACCATTCACCTCGCACTT	NM_030826.4
Cat	GACATCGGCACATGAATGGC	GTGCAAGTCTTCCTGCCTCT	NM_012520.2
Nfkb1	GACAACTATGAGGTCTCTGGGG	CTGGATCACTTCAATGGCCTC	NM_001276711.2
Nrf2	CAGCTACTCCCAGGTTGCCC	ATAGCTCCTGCCAAACTTGCT	NM_001399173.1
TOP2β	CCTGCATCAACTGGCAAACC	CACAGGCTCTGCTTCTTCCA	NM_001100858.2
Nqo1	AAGGGTGGAAGAAGCGTCTG	GATCTGGTTGTCGGCTGGAA	NM_017000.3
Pgk1	AGCGGCTGTCAGAGAAAGAG	CCTTCGACCTCTCGGATTTAG	NM_053291.3
Gucy1a1	TGAACCAGCCCTATTTGCTC	CTTGTTCACCAGCCTTC	NM_017090.2
Gucy1b1	CGAGCAAAGTCCTCAACCTC	AATGAGCCCTTTGCCTTTTT	XM_039101764.1
Hmox1	AGGATTTGTCCGAGGCCTTG	CTGGGTTCTGCTTGTTTCGC	NM_012580.2
ZIP14	ATCCAGAATCTTGGCCTCCT	AGGCAGTCTGTGCCAGTTTT	NM_001107275.1
Fth1	AAGTGCGCCAGAACTACCAC	CTCAGCATGTTCCCTCTCTTC	NM_012848.2
mfrn1	GAAGTGGTTCCGGCATCTT	TTTCTCCTGCCTACCACCAC	NM_001013996.1
fpn1	GGACTGGCTCAGCTTTCTTG	CAGGCTGACGGAGATTATGG	NM_133315.2
Abcb8	ACACTGATGCTGGCTGTTGT	TGCTTGATAGCGTTCCTCCT	NM_001007796.2
Mcoln1	CTCCAGACACGGAGACAACA	ACCAGCCATTGACAAACTCC	NM_001105903.1
Nramp2	AATGAGCAGGTGGTTGAGGT	GCGACCATTTTAGGTTCAGG	NM_001399169.1
Tfr1	GGATGAGACCAGAAGCCAAA	TCAGTGCCAAAAACGACAGA	NM_022712.1
IL-1β	ACCCAAGCACCTTCTTTTCCTT	TGCAGCTGTCTAATGGGAACAT	NM_031512.2
CD68	CACTTGGCTCTCTCATTCCCT	GCTGAGAATGTCCACTGTGCT	NM_001031638.1
CD86	GTCAAGACATGTGTAACCTGCACC	ACGAGCTCACTCGGGCTTAT	NM_020081.2
CD163	ATCACAGCATGGCACAGGT	TCCAGATCATCCGTCTTCG	NM_001107887.1
CD206	TCCCTCAATGGAACACACACTC	TTAAAAATTGCCGTGAGTCCAAGAG	NM_001106123.2
Tbp	TGGGATTGTACCACAGCTCCA	CTCATGATGACTGCAGCAAACC	NM_001004198.1
Rpl13A	GGATCCCTCCACCCTATGACA	CTGGTACTTCCACCCGACCTC	NM_173340.2

Primer sequences are given in their 5′-3′ orientation. Abcb8, ATP binding cassette subfamily B member 8. Bax, BCL2 associated X. Bcl2, B-cell lymphoma 2. Casp3, caspase 3. Cat, catalase. CD206, mannose receptor C-Type I. Col1a2, collagen type I alpha 2 chain. Col3a1, collagen type III alpha 1 chain. fpn1, ferroportin-1. Fth1, ferritin heavy chain 1. Gpx1, glutathione peroxidase 1. Gucy1a1, guanylate cyclase 1 soluble subunit alpha 1. Gucy1b1, guanylate cyclase 1 soluble subunit beta 1. Hmox1, heme oxygenase 1. IL-1β, interleukin 1 beta. IL-6, interleukin 6. Lox, lysyl oxidase. Mcoln1, mucolipin 1. mfrn1, mitoferrin-1. Nfkb1, nuclear factor kappa B subunit 1. Nox2, NADPH oxidase 2. Nox4, NADPH oxidase 4. Nqo1, NAD(P)H quinone dehydrogenase 1. Nramp2, natural

resistance-associated macrophage protein 2. Nrf2, nuclear factor erythroid-2 related factor 2. Pgk1, phosphoglycerate kinase 1. RAGE, receptor for advanced glycation end products. Rpl13A, ribosomal protein L13A. Sod1, superoxide dismutase 1. Sod2, superoxide dismutase 2. Tbp, TATA box binding protein. Tfr1, transferrin receptor 1. Tgfb1, transforming growth factor beta 1. TOP2β, DNA topoisomerase II beta. Tp53, tumor protein p53. ZIP14, Zrt- and Irt-like protein 14.

Table S2: LV echocardiographic parameters and plasma BNP at baseline.

	CTRL	DOX	DOX+PM	CTRL+PM
Conventional echocardiography				
LVEF (%)	86.7 ± 0.9	87.4 ± 1.2	85.6 ± 1.4	85.1 ± 1.6
Longitudinal LVFS (%)	24.6 ± 1.5	26.2 ± 0.8	25.6 ± 1.2	24.3 ± 1.2
Radial LVFS (%)	55.2 ± 1.8	54.1 ± 1.8	53.9 ± 1.8	52.7 ± 1.9
LV cardiac index (ml/min/m^2)	0.17 ± 0.01	0.18 ± 0.01	0.17 ± 0.01	0.17 ± 0.01
LVESV/BSA (μl/cm^2)	0.074 ± 0.007	0.075 ± 0.008	0.078 ± 0.008	0.094 ± 0.013
LVEDV/BSA (μl/cm^2)	0.553 ± 0.022	0.586 ± 0.019	0.546 ± 0.016	0.611 ± 0.027
LVSV index (μl/cm2)	0.48 ± 0.02	0.51 ± 0.02	0.47 ± 0.01	0.52 ± 0.02
LV sphericity index	0.20 ± 0.01	0.21 ± 0.01	0.18 ± 0.01	0.20 ± 0.01
$LVAW_d$ (mm)	1.88 ± 0.07	1.76 ± 0.04	1.71 ± 0.06	1.70 ± 0.04
$LVPW_d$ (mm)	1.81 ± 0.04	1.75 ± 0.06	1.83 ± 0.06	1.77 ± 0.07
E/A	1.38 ± 0.05	1.52 ± 0.07	1.38 ± 0.04	1.37 ± 0.04
E/E'	−33.2 ± 2.1	−28.7 ± 1.9	−31.1 ± 1.5	−29.6 ± 2.2
HR (bpm)	351.9 ± 9.9	358.7 ± 10.8	360.7 ± 8.0	344.0 ± 8.6
BSA (cm^2)	334.3 ± 7.8	337.6 ± 11.0	333.5 ± 8.6	329.8 ± 7.1
Strain				
LVGLS (%)	−40.7 ± 1.8	−39.6 ± 1.9	−35.9 ± 1.9	−36.1 ± 2.1
LVGCS (%)	−40.2 ± 1.2	−39.9 ± 0.9	−37.0 ± 1.4	−37.8 ± 1.2
LV peak radial strain (%)	105.3 ± 8.6	97.3 ± 4.1	84.3 ± 7.2	87.7 ± 5.9
Plasma				
BNP (ng/ml)	0.11 ± 0.02	0.11 ± 0.02	0.14 ± 0.02	0.15 ± 0.02

LV echocardiographic parameters and plasma BNP were measured in CTRL (N=14), DOX (N=15), DOX+PM (N=18), and CTRL+PM (N=14) animals at baseline. Random missing values are due to poor image quality. For plasma BNP: CTRL (N=12), DOX (N=15), DOX+PM (N=17), and CTRL+PM (N=13). Data are presented as mean ± SEM. A, mitral flow velocity in the late filling phase. BNP, brain natriuretic peptide. BSA, body surface area. DOX, doxorubicin. E, mitral flow velocity in the early filling phase. E', peak septal mitral annulus velocity in the early filling phase. HR, heart rate. LV, left ventricular. $LVAW_d$, LV anterior wall in diastole. LVEF, LV ejection fraction. LVEDV, LV end-diastolic volume. LVESV, LV end-systolic volume. LVFS, LV fractional shortening. LVGCS, LV global circumferential strain. LVGLS, LV global longitudinal strain. $LVPW_d$, LV posterior wall in diastole. LVSV, LV stroke volume. PM, pyridoxamine.

Table S3: RV Hemodynamic parameters and lung wet-to-dry weight ratio at week 8.

	CTRL	DOX	DOX+PM	CTRL+PM
Hemodynamic parameters				
RVESP (mmHg)	25.5 ± 1.5	22.8 ± 1.2	26.8 ± 1.7	29.1 ± 0.0
RVEDP (mmHg)	1.5 ± 1.9	3.1 ± 0.7	6.3 ± 1.2	4.4 ± 1.0
RV dP/dt_{max} (mmHg/s)	1835.0 ± 90.8	1403.0 ± 139.9	1493.0 ± 126.5	1914.0 ± 254.5
RV dP/dt_{min} (mmHg/s)	−1475.0 ± 214.0	−1276.0 ± 89.7	−1222.0 ± 115.7	−1739.0 ± 156.3
RV contractility index (1/s)	146.0 ± 14.6	113.9 ± 11.7	96.3 ± 10.7	137.1 ± 24.3
Organ weight				
Lung wet-to-dry weight ratio	5.03 ± 0.09	5.41 ± 0.16	5.27 ± 0.10	4.95 ± 0.09

RV hemodynamic parameters and lung wet-to-dry weight ratio measured in CTRL (N=3), DOX (N=7), DOX+PM (N=7), and CTRL+PM (N=3) animals at sacrifice. For lung wet-to-dry weight ratio: CTRL (N=14), DOX (N=13), DOX+PM (N=18), and CTRL+PM (N=14). Data are presented as mean ± SEM. DOX, doxorubicin. PM, pyridoxamine. RV, right ventricle. RV dP/dtmax, maximum peak time derivative. RV dP/dtmin, minimum peak time derivative. RVEDP, right ventricular end-diastolic pressure. RVESP, right ventricular end-systolic pressure.

Supplementary Figures

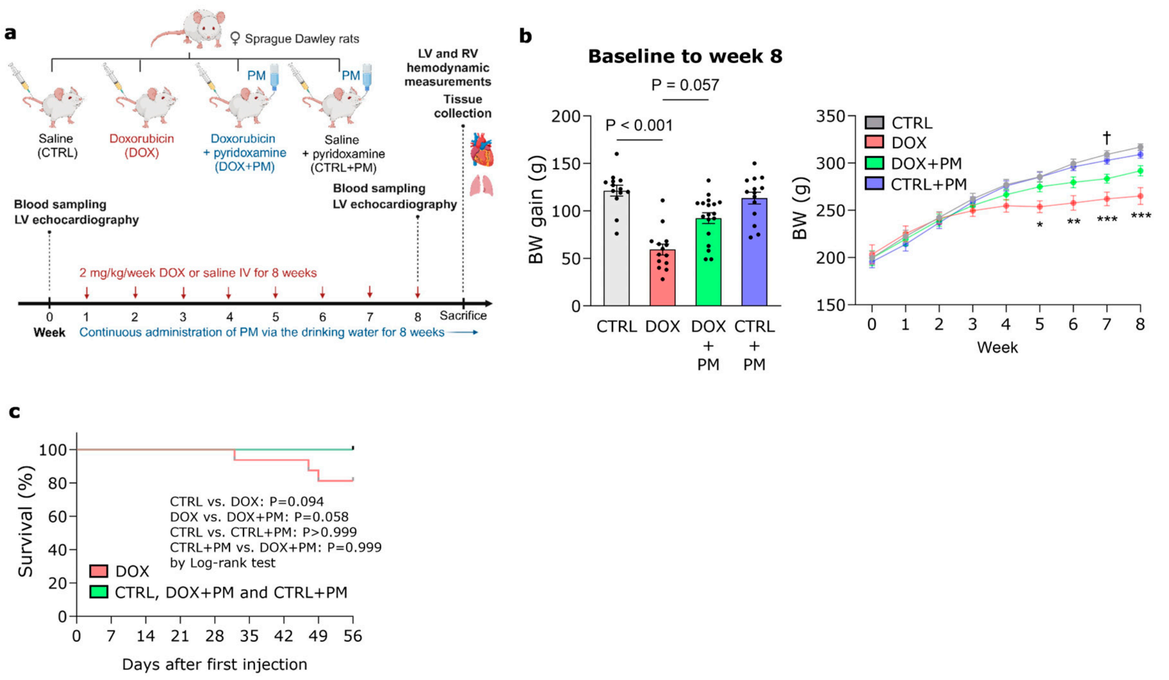

Figure S1: Study design, BW, and survival. **(a)** Parallel group design consisting of female Sprague Dawley rats randomly divided into four groups: CTRL (N=14), DOX (N=15), DOX+PM (N=18), and CTRL+PM (N=14). DOX (2 mg/kg) or 0.9% saline was administered weekly intravenously, denoted by black arrows. PM (1 g/L) was

administered *ad libitum* via drinking water from the first injection. BW was measured weekly. Echocardiography was performed at baseline and week 8, together with blood sampling. Invasive hemodynamic measurements of the LV and RV were performed at sacrifice. The heart and lungs were isolated and weighed. **(b)** BW gain from baseline to week 8 (left panel) and progression of bodyweight over time (right panel) for all groups for CTRL (N=13), DOX (N=14), DOX+PM (N=18), and CTRL+PM (N=14) animals. **(c)** Survival for all groups represented as a Kaplan-Meier plot. For B, right: $^{*}P<0.05$, $^{**}P<0.01$ and $^{***}P<0.001$ vs. CTRL and $^{+}P<0.05$ DOX+PM vs. CTRL+PM. Figure A is created with BioRender.com. BW, body weight. DOX, doxorubicin. LV, left ventricular. PM, pyridoxamine. RV, right ventricular.

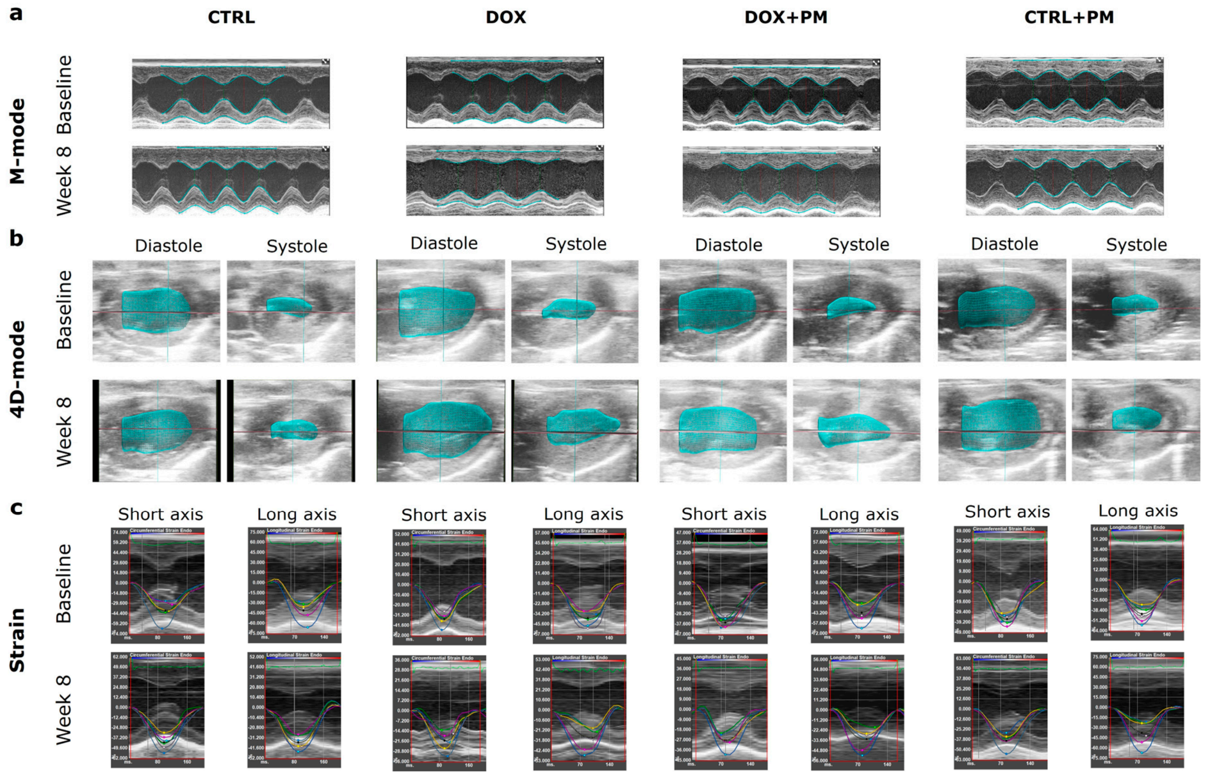

Figure S2: Representative echocardiographic images. **(a-c)** Representative pictures of M-mode obtained in parasternal short-axis view **(a)**, 4D reconstructed hearts during diastole and systole **(b)**, and circumferential and longitudinal strain in parasternal short-axis and long axis view **(c)** at baseline and week 8 of CTRL, DOX, DOX+PM, and CTRL+PM animals.

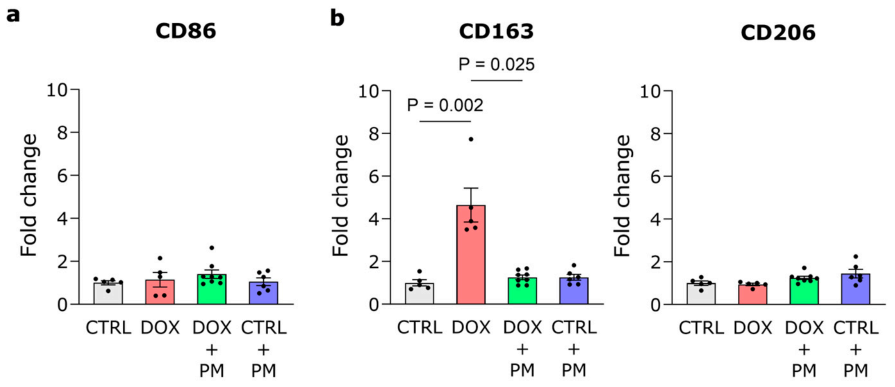

Figure S3: Expression of macrophage genes in cardiac LV tissue. **(a-b)** Quantification of the gene expression of the pro-inflammatory macrophage marker CD86 **(a)** and anti-inflammatory macrophage markers CD163 and CD206 **(b)** in LV tissue from CTRL (N=5), DOX (N=5), DOX+PM (N=8), and CTRL+PM (N=6) animals. LV, left ventricular.

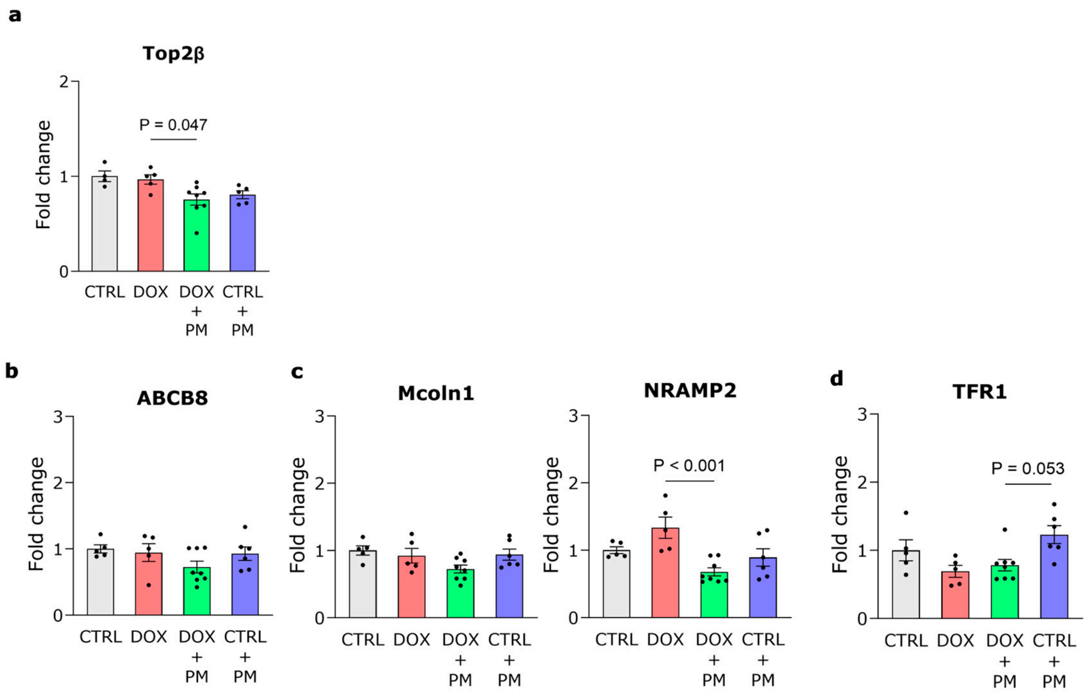

Figure S4: Expression of TOP2β gene and iron metabolism genes in cardiac LV tissue. **(a)** Quantification of DNA TOP2β gene expression in LV tissue from CTRL (N=4), DOX (N=5), DOX+PM (N=8), and CTRL+PM (N=5) animals. **(b-d)** Quantification of the expression of the mitochondrial iron exporter gene ABCB8 **(b)**, endosomal iron exporter genes Mcoln1 and NRAMP2 **(c)**, and transferrin receptor gene involved in iron import TFR1 **(d)** in LV tissue from CTRL (N=5), DOX (N=5), DOX+PM (N=8), and CTRL+PM (N=6) animals. DOX, doxorubicin. LV, left ventricular. PM, pyridoxamine.
